# Phenotypic and environmental correlates of natal dispersal in a long-lived territorial vulture

**DOI:** 10.1038/s41598-021-84811-8

**Published:** 2021-03-08

**Authors:** David Serrano, Ainara Cortés-Avizanda, Iñigo Zuberogoitia, Guillermo Blanco, José Ramón Benítez, Cecile Ponchon, Juan Manuel Grande, Olga Ceballos, Jon Morant, Eneko Arrondo, Jabi Zabala, Eugenio Montelío, Enrique Ávila, José L. González, Bernardo Arroyo, Óscar Frías, Erick Kobierzycki, Rafael Arenas, José Luis Tella, José Antonio Donázar

**Affiliations:** 1 Dept Conservation Biology, Estación Biológica de Doñana (EBD-CSIC), Américo Vespucio 26, 41440 Sevilla, Spain; 2 Animal Ecology and Demography Unit, IMEDEA (CSIC-UIB), Miquel Marquès 21, 07190 Esporles, P. Mallorca Spain; 3 Estudios Medioambientales Icarus S.L., San Vicente 8. 6ª Planta. Dpto 8. Edificio Albia I., 48001 Bilbao, Spain; 4 Dept of Evolutionary Ecology, Museo Nacional de Ciencias Naturales (MNCN-CSIC), José Gutiérrez Abascal 2, 28006 Madrid, Spain; 5 Agencia Medioambiente & Agua, Linea Geodiversidad & Biodiversidad, 41092 Sevilla, Spain; 6 Conservatoire d’espaces naturels Provence-Alpes-Côte d’Azur, Maison de la Crau 2 place Léon Michaud, 13310 Saint Martin de Crau, France; 7 ColBEC, INCITAP-CONICET-UNLPam/FCEyN-UNLPam, Uruguay 151, Santa Rosa 6300, La Pampa, Argentina; 8 UGARRA, Carlos III 1, 31002 Pamplona, Spain; 9 Department of Ornithology, Aranzadi Sciences Society, Zorroagagaina 11, 20014 Donostia-S. Sebastián, Spain; 10 Department of Applied Biology, Miguel Hernández University, Avda. de la Universidad s/n, 03202 Elche, Spain; 11 Department of Zoology and Animal Cell Biology, University of the Basque Country (UPV/EHU), Paseo de la Universidad 7, Vitoria‐Gasteiz, 01006 Spain; 12 Consultora CMC Sistemas de Mejora S.L., Vara de Rey, 48, Logroño, Spain; 13 19264, Alboreca, Spain; 14 64800, Bruges-Capbis-Mifaget, France; 15 Delegación Territorial Córdoba CAGPDS, Tomás de Aquino, 7 planta, 14071 Córdoba, Spain

**Keywords:** Ecology, Zoology, Ecology, Environmental sciences

## Abstract

Natal dispersal, the movement between the birth and the first breeding site, has been rarely studied in long-lived territorial birds with a long-lasting pre-breeding stage. Here we benefited from the long-term monitoring programs of six populations of Egyptian vultures (*Neophron percnopterus*) from Spain and France to study how the rearing environment determines dispersal. For 124 vultures, we recorded a median dispersal distance of 48 km (range 0–656 km). Linear models were used to assess the effect of population and individual traits on dispersal distance at two spatial scales. Dispersal distances were inversely related to vulture density in the natal population, suggesting that birds perceive the abundance of conspecifics as a signal of habitat quality. This was particularly true for declining populations, so increasing levels of opportunistic philopatry seemed to arise in high density contexts as a consequence of vacancies created by human-induced adult mortality. Females dispersed further than males, but males were more sensitive to the social environment, indicating different dispersal tactics. Both sexes were affected by different individual attributes simultaneously and interactively with this social context. These results highlight that complex phenotype-by-environment interactions should be considered for advancing our understanding of dispersal dynamics in long-lived organisms.

## Introduction

Natal dispersal, the movement between the birth and the first breeding location, usually constitutes the longest effective dispersal displacement that animals make throughout their lives^1,2^. By relocating genes and individuals across the landscape, natal dispersal affects individual fitness and population dynamics, so it has multiple evolutionary and ecological consequences^3–6^. As a key ecological process, it affects the genetic structure, geographical expansion, and the dynamics and stability of (meta)populations^4^, with profound implications for conservation and management. Despite this, our knowledge of this phenomenon is still inadequate and mostly based on indirect methods^4,5^, to the extent that many key unknowns in basic and applied ecology are directly related to a poor understanding of dispersal processes and patterns^7–10^.

Studying dispersal is complicated, so even descriptive approaches are subject to serious methodological biases. For example, dispersal patterns derived from capture-recapture data are often biased by the limited spatial scale over which movements were studied^11^. This precludes detecting long-distance dispersers, a relevant issue because they can contribute disproportionally to range shifts and population dynamics^12,13^. Despite these methodological difficulties, a pervasive pattern even among actively dispersing animals is that the frequency of dispersers decreases with the distance from the source location, with most individuals displacing short or medium distances and a minority moving long distances^2,14^.

To the difficulties of obtaining unbiased estimates must be added the fact that dispersal is a complex phenomenon that depends on multiple causes acting at different levels and scales^5,15^. Fixed dispersal strategies are rarely expected^5,16^, and it is currently accepted that animals may respond plastically to variations in the costs and benefits of dispersal over the short-term. At this proximate level, dispersal is influenced by the environmental context and by internal state variables of the individual organism (condition- and phenotype-dependent dispersal, respectively, according to^17^). Biotic and abiotic factors comprise the condition-dependent, environmentally induced causes of dispersal. For example, dispersal is a common response of organisms to population saturation or deteriorating habitat quality^17–19^. On the other hand, individual traits such as age, sex, body condition, development effects, and the behavioural type may modulate phenotype-dependent dispersal^5,17^. A widespread pattern is that dispersal is sex-biased, but there are few data or contradictory results regarding the effect of many other internal traits. This may be explained because the above-mentioned factors can be expected to interact with each other. Understanding these interactions, and in particular how the internal state of individuals interacts with the environmental context to produce different dispersal phenotypes, is of crucial interest for generating landscape-specific predictions about dispersal of individuals or whole populations. This aspect, however, has received little attention^20–22^.

Birds are prime models in the study of dispersal in animals. However, most knowledge from long-lived species with a long pre-adult period has a major bias towards colonial species. Studies on dispersal of long-lived territorial species are very scarce and mostly descriptive (e.g.^23^) or restricted to particular stages of the dispersal process (e.g.^24^). Here we benefited from several long-term marking and monitoring programs of a territorial bird with a long-delayed onset of reproduction, the Egyptian vulture *Neophron percnopterus*, to describe large-scale natal dispersal patterns and examine the potential determinants of dispersal distances. Particularly, we investigated the influence of internal and external factors on individual variation in natal dispersal, and how these different forces combine and interact to explain dispersal patterns.

### Hypotheses and predictions

Our research framework is based on the idea that the dispersal phenotypes are the result of the different sensitivity of individuals to environmental cues and constraints experienced in the natal patch^25^. Phenotype-by-environment interactions may modulate dispersal in complex and often unpredictable manners (e.g.^21,22^), but it may be practical to review some general predictions to help understand how these interactions may change the strength and direction of single effects.

Most empirical studies report positive density-dependent dispersal^26^, with individuals being more prone to disperse and/or moving further the higher the density of conspecifics, due to resource depletion (exploitative competition) and/or direct competitive interactions (interference competition). Conversely, the opposite view of individuals dispersing less from high-density areas has also found some support, arguably because conspecifics may be used as cues of environmental quality^27^. Density-dependent approaches are, however, often founded on the assumption that density informs on habitat quality and saturation, but this does not necessarily have to be the case^28^. Density values can have different meanings, so complementary data on individual behaviour and habitat quality may be key to separate social effects from other confounding factors when experimental approaches are unfeasible^15,26,29^. For example, if individuals are able to perceive deteriorating habitat conditions, they may tend to escape from declining populations by moving further^30^, irrespective of population density. Alternatively, it could be hypothesized that average dispersal distances would be longer from stable populations because of the fewer unoccupied sites to settle and the higher competitive context for these locations in the natal area.

Among internal state variables, sex-biased dispersal is pervasive in socially monogamous birds^31,32^, so we predicted that this should hold true for Egyptian vultures. As both sexes seem to have different functions in territory acquisition and defence, and because female-biased adult mortality has been reported in Spain^33^, we predict that the effect of density and competition on dispersal should be higher in males. In such a long-delayed breeder with a large variance in age of first reproduction^33^, dispersal distance could also be expected to vary with recruitment age. In species with delayed breeding, several non-exclusive hypotheses have been proposed to explain why some individuals hold a territory as soon as they reach sexual maturity while others remain as floaters well beyond this age^34^. They can be summarized in (1) constrain hypotheses, which postulate that some individuals are inferior and cannot hold a territory until they acquire the necessary age, experience, and skills^35^. If individuals are constrained in such a way, we would expect young recruits to be of higher quality, thus dispersing shorter distances from high-quality natal areas; (2) restraint hypotheses, which postulate that early reproduction implies future fitness costs in terms of survival and/or breeding prospects^36^. In this way, some individuals would recruit as soon as they can obtain a breeding vacancy whatever its quality, while others would voluntarily delay breeding to acquire dominance status while queueing for a high-quality territory. Under this hypothesis, birds with a delayed breeding strategy would recruit at shorter distances than early recruits whenever the natal area is perceived as high quality. Other phenotypic traits, mediated by, and interacting with environmental and/or parental effects may determine natal dispersal distances. Dispersal is known to entail costs during transience and settlement stages because individuals have to cope with unfamiliar environments in which they must find food, avoid various sources of mortality, and secure a territory and a mate^37^. Philopatry to the natal area may be the most advantageous strategy in these circumstances, so we predicted a negative relationship between natal phenotypic traits presumably conferring competitive advantages and dispersal distance. Factors such as date of birth, the hierarchy within the natal brood, or body condition may reflect these natal conditions and may determine dispersal patterns^21,38–40^. However, the benefits of emigrating from poor or deteriorating patches may overcome the fitness costs of dispersal, particularly in bearers of phenotypic traits that reduce dispersal costs^17^, so in this situation superior individuals are likely to disperse further^20^. Dispersal can also be an efficient way to avoid competition with kin (reviewed in^5^). Brood size could be a proximate cue informing on sib-competition, so longer dispersal distances could be expected in vultures from double-chick broods.

## Results

### Dispersal patterns

**Figure 1:**
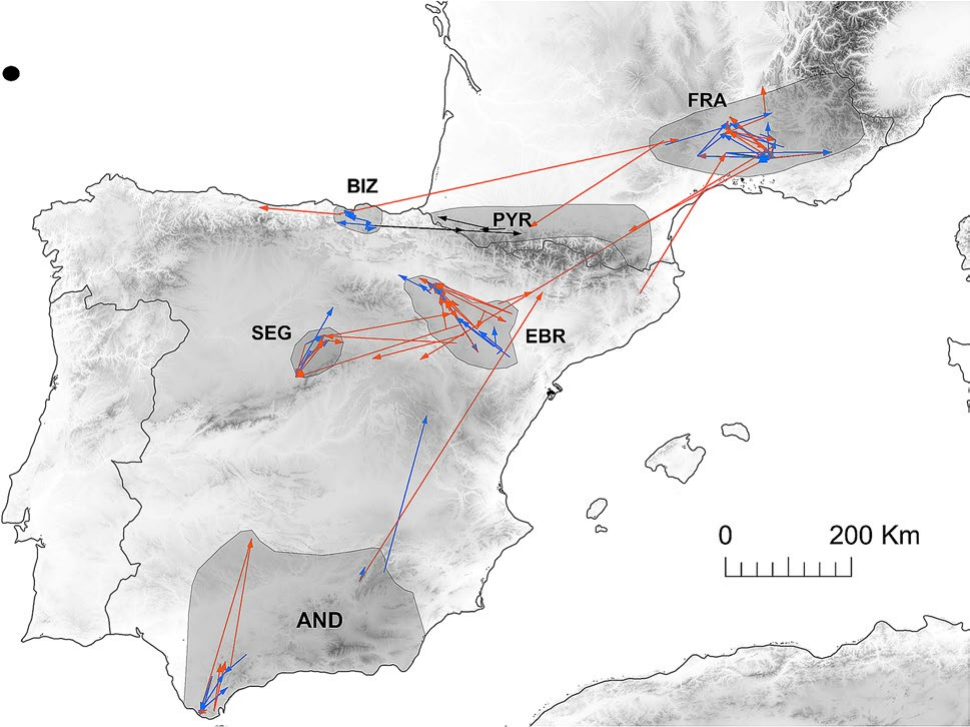
Euclidean distances between the natal and the first-breeding site of Egyptian vultures in Spain and France. Blue: males; Orange: females; Black: non-sexed birds. The distribution of the study populations is also shown. *AND* Andalusia, *BIZ* Bizkaia, *EBR* Ebro Valley, *FRA* France Southeast, *PYR* France Pyrenees, *SEG* Segovia. The background map was generated by DS modifying and assembling the digital elevation models available at the Copernicus Land Monitoring Service (https://land.copernicus.eu/imagery-in-situ/eu-dem), using ArcGIS 10.2 (https://www.esri.com/en-us/arcgis).

**Figure 2:**
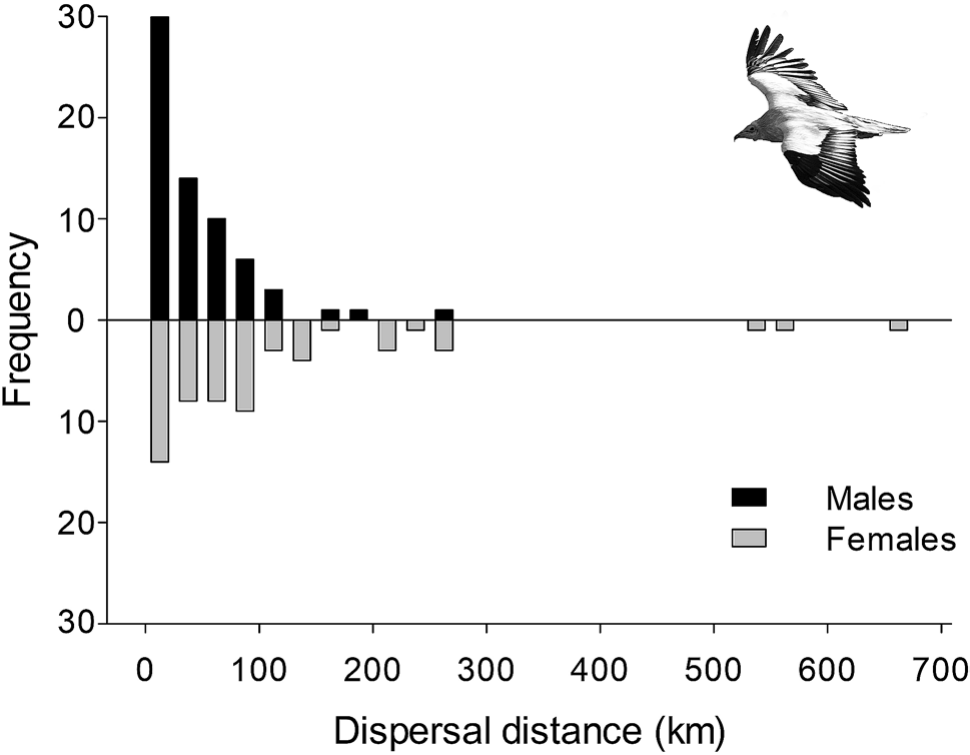
Frequency distribution of natal dispersal distances of Egyptian vultures in Spain and France. Photo credit Egyptian vulture: D. Serrano.

At the regional scale, we had data on natal dispersal of 124 vultures (66 males and 58 females). The vast majority of birds dispersed to a greater or lesser extent from the natal territory to establish their own breeding territories, but three males recruited in their natal nest (Fig. 1). Dispersal distances were highly leptokurtic (Kurtosis = 13.2) and fat-tailed, especially in females (Fig. 2). Median dispersal distance was 48 km (interquartile range IQR 16–91.8 km, range 0–656 km), with females moving on average almost three times further than males (females: median 71.7 km; IQR 28.6–131.2, range 6–656; males: median 28.5, IQR 9.9–61.6, range 0–260.6).

At the local scale of the Ebro valley, we recorded the dispersal distances of 41 birds (23 males and 18 females) with a median distance of 17.1 km, (IQR 6.6–36.9, range 0–134.9 km). Again, females tripled the distances covered by males (females: median 28.7 km; IQR 15.5–58.5, range 8.2–134.9 km; males: median 9.7 km, IQR 3.6–24.2, range 0–98).

### Correlates of natal dispersal

**Figure 3:**
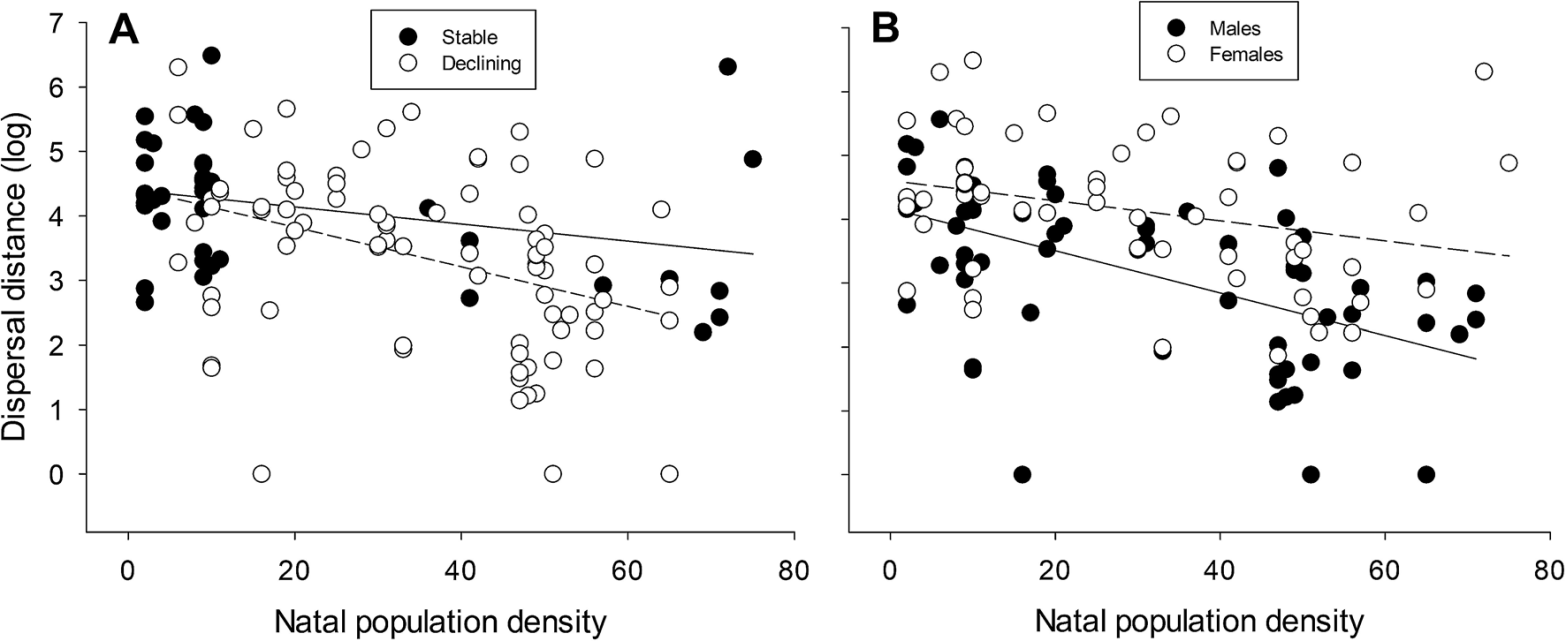
(**A**) Relationship between dispersal distance (log scale) and natal population density (number of breeding pairs in a buffer of 48 km around the natal nest). Trends from stable/increasing populations (filled circles, solid line) and decreasing populations (open circles, dashed line) are shown separately. (**B**) Relationship between dispersal distance (log scale) and natal population density (number of breeding pairs in a buffer of 48 km around the natal nest). The solid and the dashed line correspond to males and females respectively.

Linear models for the whole dataset revealed clear support for the effect of sex, breeding population density, and population trends at the population of origin on natal dispersal distances, as these variables were always included as main effects in the 95% confidence set of models (Table S1). In general, females dispersed longer distances than males, and birds moved shorter distances the higher the breeding population density and when they originated from declining populations (Table 1). Two interactions were included among the top-ranked models as strongly supported by data: (1) models including the interactive effect of density and population trends were 10 times more supported than models without this effect according to the evidence ratio of Akaike weights, indicating that the negative density-dependent effect on dispersal distance was more pronounced in declining populations (Fig. 3A); (2) models including an interaction between density and sex were 5 times more supported than models including only the corresponding additive effects, suggesting that the effect of density on natal dispersal distance was stronger for males than for females (Fig. 3B). More uncertain was the effect of age at recruitment on dispersal distance, as models incorporating this predictor, often with an interaction with sex, were only a bit more supported by data than models without it (Akaike weights of 0.63 and 0.32, respectively, Table S1).

**Table 1:** Conditional model averaging of the top-ranked models (delta AICc < 2), examining the correlates of natal dispersal distances of Egyptian vultures at the regional (whole study area, Spain and France) and local scale (Ebro valley).

Parameters	Estimate	Adj. SE	Lower	Upper	RVI
**Regional scale**					
Intercept	**3.722**	**0.100**	**3.525**	**3.918**	
Sex (females)	**1.034**	**0.191**	**0.659**	**1.408**	1
Density	**− 0.024**	**0.005**	**− 0.034**	**− 0.014**	1
Trend (stable)	**0.635**	**0.219**	**0.206**	**1.063**	1
Age	**− **0.026	0.031	**− **0.086	0.034	0.62
Sex × density	**0.021**	**0.009**	**0.003**	**0.039**	1
Sex × age	0.094	0.061	**− **0.025	0.213	0.25
Density × trend	**0.024**	**0.010**	**0.005**	**0.043**	1
Age × density	**− **0.002	0.001	**− **0.005	0.001	0.14
**Local scale**					
Intercept	**2.894**	**0.257**	**2.390**	**3.398**	
Sex (females)	**1.256**	**0.310**	**0.647**	**1.864**	1
Density	**− 0.047**	**0.012**	**− 0.071**	**− 0.023**	1
Age	**− **0.040	0.042	**− **0.124	0.043	0.74
Brood size	**− **0.296	0.308	**− **0.900	0.308	0.09
Body condition	3.683	2.851	**− **1.905	9.270	0.37
Sex × age	0.145	0.090	**− **0.032	0.321	0.42

Model average parameter estimates, adjusted standard errors, 95% confidence intervals and relative variable importance (RVI, the sum of Akaike weights over the set of models in which the variable appears) are shown. Effects with 95%CI not overlapping zero are shown in bold.

**Figure 4:**
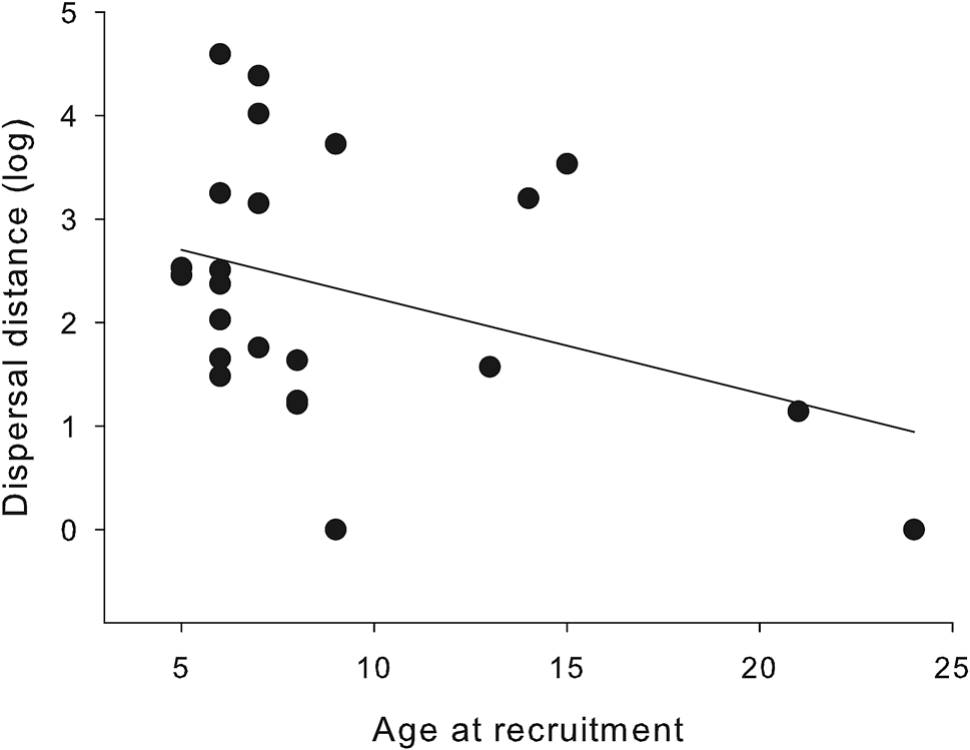
Relationship between dispersal distance (log scale) and age at recruitment for Egyptian vulture males at the local scale (Ebro valley).

**Figure 5:**
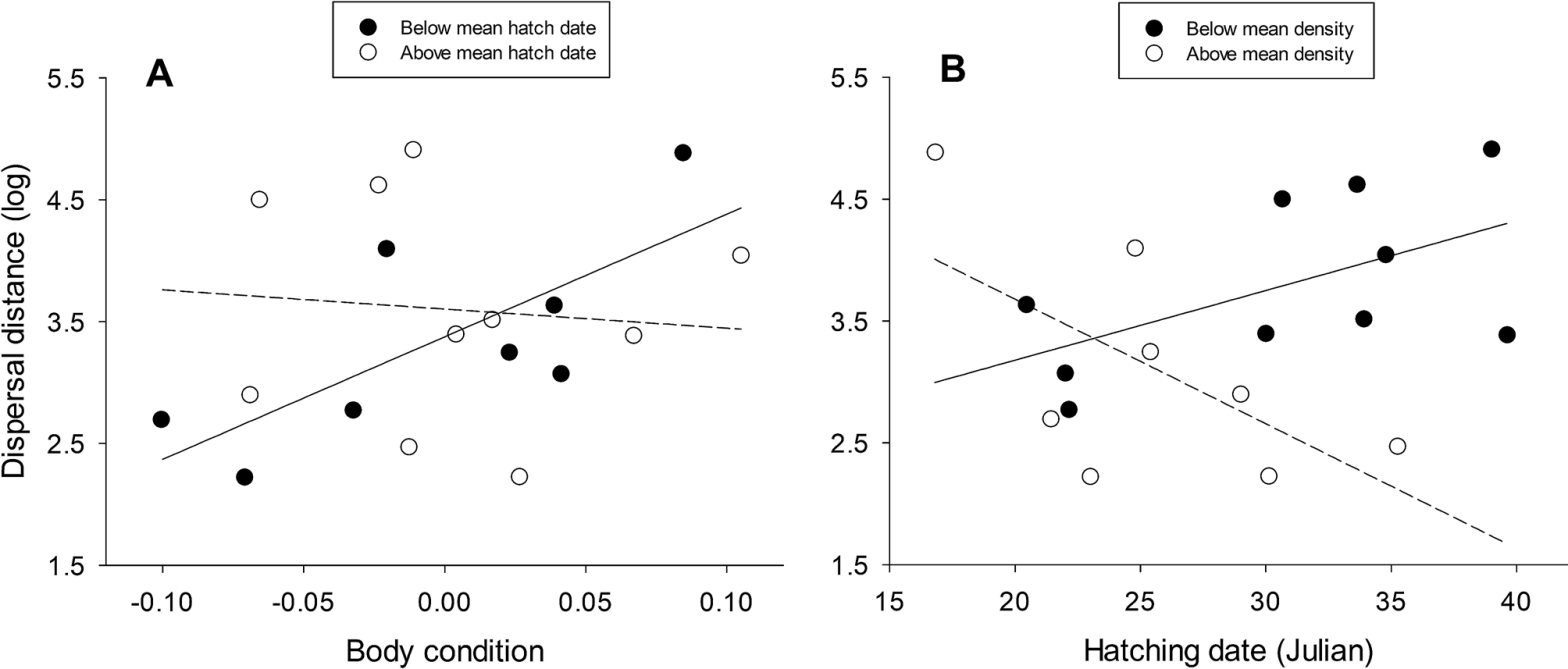
Relationship between dispersal distance (log scale) and body condition for Egyptian vulture females hatched early and late (**A**) and between dispersal distance and hatching date for females born at low and high densities (**B**) at the local scale (Ebro valley).

Results at the Ebro valley local scale were congruent with those found at the regional scale (Table S2), but effect sizes were, in general, lower. Model average estimates clearly supported female-biased dispersal distances and a negative effect of density, but the effect of age was again uncertain (Table 1). When we built models separately for each sex, we found a complex interplay between variables that affected males and females differently (Tables S3 and S4). For males, model-averaged estimates indicated negative relationships between dispersal distance and density and between dispersal distance and age (Table 2, Fig. 4). In the case of females, hatching date, body condition, and density affected dispersal distance in a complex manner (Table 2). Thus, among early hatched females, dispersal distances tended to increase with body condition, while there was no apparent trend with body condition among late-hatched birds (Fig. 5A). Females born in dense conspecific environments, in turn, moved shorter distances the later they hatched, while the opposite seemed to occur for females from low-density areas (Fig. 5B).

**Table 2:** Conditional model averaging of the top-ranked models (delta AICc < 2), examining the correlates of natal dispersal distances of male and female Egyptian vultures at the local scale (Ebro valley).

Parameters	Estimate	Adj. SE	Lower	Upper	RVI
**Males**					
Intercept	**2.345**	**0.229**	**1.896**	**2.794**	
Density	**− 0.054**	**0.018**	**− 0.089**	**− 0.019**	1
Age	**− 0.090**	**0.045**	**− 0.179**	**− 0.002**	0.81
Body condition	3.947	4.212	**− **4.307	12.202	0.18
Density × age	**− **0.005	0.004	**− **0.013	0.004	0.17
Density × body condition	**− **0.642	0.350	**− **1.329	0.044	0.18
**Females**					
Intercept	**3.165**	**0.186**	**2.802**	**3.529**	
Density	**− **0.013	0.017	**− **0.047	0.021	1
Hatching date	0.025	0.029	**− **0.033	0.082	1
Body condition	3.311	2.628	**− **1.839	8.461	0.67
Density × hatching date	**− 0.012**	**0.004**	**− 0.020**	**− 0.004**	1
Hatching date × body condition	**− 0.904**	**0.392**	**− 1.672**	**− 0.136**	0.67

Model average parameter estimates, adjusted standard errors, 95% confidence intervals and relative variable importance (RVI, the sum of Akaike weights over the set of models in which the variable appears) are shown. Effects with 95%CI not overlapping zero are shown in bold.

Model diagnostic checking indicated reasonable agreement with the observed data (Figs. S1 and S2). The residuals of the highest-ranked models obtained at the two scales did not show spatial autocorrelation according to Moran’s I and semiovariograms (Table S5 and Fig. S3).

## Discussion

We investigated natal dispersal patterns and their potential determinants in a long-lived vulture with a prolonged pre-breeding stage. Our large scale analyses involving populations with contrasting densities and trends allowed us to capture wide spatial variation in dispersal distances and environmental conditions. We found that dispersal distance was inversely density-dependent, particularly in declining populations. We also found that some internal state variables influenced dispersal distances, but often interacting with the social environment and differently in each sex. These results show that a complex interplay between phenotypic traits and the natal environmental context determine what types of individuals disperse and how far they move^5,15,17,25^.

The general tendency of Egyptian vultures for being philopatric at the landscape scale occurred in spite of their long-distance migratory movements and long and wide-ranging nomadic pre-breeding lifestyle. This can be explained in evolutionary terms by the simple fact that being born in a particular place informs that this place is of sufficient quality for the species to breed^41^. Other selective advantages of philopatry are the preservation of co-adapted genomes^42^, and the familiarity with the natal environment^3^. Whatever the ultimate mechanism, this result agrees with the prevailing view of distance-decaying kernels in virtually all organisms^13^, and in birds in particular^2^. At the same time, there was substantial individual variation. Previous information on long-distance dispersal in Egyptian vultures was anecdotic^43^, so dispersal has been assumed to be of much lower magnitude in studies of population dynamics^44–47^. Our estimates of dispersal at the regional scale were much larger than those derived from the movement of birds at the local scale, remarking the importance of considering appropriate spatial scales in dispersal studies^11^. Although we did not monitor the whole breeding population of Spanish and French Egyptian vultures, and certainly some individuals were lost from observation, our large-scale approach at the regional scale minimizes this kind of sampling bias distortion. Indeed, natal dispersal patterns found at this scale seem more realistic at explaining two features of the spatial structure and dynamics of Egyptian vultures in southern Europe. First, the dynamics of semi-isolated populations have been shown to be strongly affected by immigration^48^, which necessitates some level of medium to long-distance dispersal. Second, our findings concur with the genetic panmixia described for the Spanish mainland population, but with a certain signature of isolation in the very peripheral population of Southern Spain^49^.

### Correlates of dispersal

From a more mechanistic perspective, our results show the importance of the demographic context on the distance moved by dispersers. Contrary to the most general finding of positive density-dependent dispersal patterns in territorial animals^26^, we found that birds moved shorter distances the highest the density of breeding conspecifics in the surroundings of the natal site. This also contrasts with most theoretical models of dispersal evolution, which predict positive density-dependent dispersal in competitive scenarios^50–53^. However, negative density-dependency in natal dispersal has been found in some species of territorial birds^54–56^ and other vertebrates^26^, and could be explained because birds born in dense environments could perceive a higher chance of finding a vacancy and/or a mate in the surroundings of the natal location^57^. Moreover, the abundance of conspecifics could be used as a reliable indicator of habitat quality^27^, modulating the propensity of individuals to abandon the natal area^58^. Consistent with these views, we detected a strong interactive effect of conspecific density around the natal patch and population trend, so that shorter dispersal distances occurred in birds born in dense but declining populations where more vacancies are available.

This last result does not support the prediction of longer dispersal distances from deteriorating populations, and is probably related to the fact that the main cause of population regression in most Egyptian vulture populations of Western Europe is human-induced adult mortality, with poisoning as the predominant factor^44,46,59^. Adult mortality and territorial extinction create vacant sites in the population which would facilitate individual recruitment closer to the natal territory^60–62^. However, contrarily to other demographic processes which may signal habitat degradation such as the quantity and quality of offspring raised by conspecifics^63^, birds are probably incapable of perceiving changes in habitat quality associated with an increase in anthropogenic adult mortality^64^. As breeding success in these declining populations is not higher than in stable or increasing ones^46,47,65,66^, our results suggest that settlement patterns of first breeders could be conditioned at least in part by habitat selection behaviours leading to an incorrect perception of habitat quality (ecological traps^67,68^). Conspecific attraction may result in this type of maladaptive behaviour if conspecifics are no longer valid indicators of habitat quality^69^, particularly if coupled with adult site fidelity in what has been called “social inertia”^70^.

We also found that birds respond to the natal social environment in different ways depending upon individual traits such as sex, age, and the conditions experienced during the rearing stage. Egyptian vulture females dispersed on average longer distances than males, and indeed in our data set the three birds that recruited in the nest of birth were males, while the three cases of movement distances longer than 500 km corresponded to females. This result is in accordance with the wealth of previous empirical evidence of females being more dispersive than males in birds^31,32^, and in raptors in particular^71^, supporting a general selective advantage for males to stay closer to their natal sites. Several mostly non-exclusive hypotheses have been proposed to explain these differences, although the resource competition hypothesis proposed by Greenwood^31^ would be the most supported by data^72^. Under this hypothesis, males of territorial monogamous birds often acquire and defend local resources, so they would benefit more from being philopatric due to familiarity with the natal area, while females would invest in searching for males in larger areas and eventually disperse further. Moreover, locating and defending a territory is an intensive task that would prohibit males from sampling extensive areas rapidly, while females would be more free to prospect wide areas in search of males^73^. The fact that conspecific density was particularly influential on the dispersal distance of males, with females being more insensitive to this factor, would support this hypothesis (see also^57^). Contrary to other studies of long-lived birds^39,74–76^, we found evidence that dispersal distance diminishes with recruitment age in Egyptian vultures, particularly in males. Recruitment age could be biased by detection probability, for example if breeders are not detected in the year of recruitment. However, resighting rates of breeders were very high in our populations^48,77^, and once recruited Egyptian vultures show extreme fidelity to their territories (only 2.2% of 589 breeding attempts in consecutive years produced a territory change, median distance = 7 km, range = 0.7–12.5 km). Moreover, our findings of a negative relationship between dispersal distance and recruitment age were contrary to what would be expected under distance-decaying resighting rates^78^. Hence, our estimates are probably very close to reality. Since birds dispersed shorter distances when there were many conspecific breeders in the surroundings, our findings could be related to the simultaneous coexistence of different life-history tactics as to when and where to recruit, two decisions that probably cannot be separated^79^. Sexually mature birds from dense populations could prefer to delay breeding, gaining skills, experience, and local site dominance while queueing for a territory in the surroundings of the natal territory or, alternatively, try to settle as soon as possible by moving away to seek less competitive environments. This hypothesis would be in accordance with the more pronounced effect of recruitment age on dispersal distance of males, the sex that presumably acquires resources, and with the biased mortality of female breeders observed in Egyptian vultures^33^, which would relax competition for breeding opportunities in this sex.

Finally, date of birth and body condition seemed to affect dispersal distances in females. Hatching date interacted with body condition, with females hatched early in the breeding season and with a good body condition dispersing the longer distances. Both variables are usually considered as good proxies of individual quality in birds, and indeed have been related to survival prospects later in life^80^, so these females probably decided voluntarily to move further. In addition, body condition may be a prerequisite for not suffering high costs during long-distance movements^81^, and may affect different aspects of the dispersal process related to landscape exploration, such as speed, tortuosity, and path length^82^. In late hatched birds, however, body condition did not appear to influence dispersal, which was shaped instead by the density of breeders in the natal population. Although the correlative nature of our data hinders identifying the precise mechanisms involved, our results indicate that the distinct developmental conditions experienced by individuals, interacting with the environmental context, have important effects on dispersal asymmetries later in life.

### Conservation prospects

Egyptian vulture populations suffered a dramatic human-induced decline in the past that made the species disappear from many locations of Spain and France^44,48^. Currently, some of the threatening causes have subsided, others continue operating, and new conservation challenges are arising, with the result that the species is recovering in certain areas^47,48,83^ while continues to decline in others^84^. In this context, relevant questions are how natal dispersal strategies may contribute to local population persistence and dynamics. Our findings suggest that Egyptian vultures, like most raptors, have evolved under conditions selecting for philopatry at a regional scale, probably due to spatial variability but temporal autocorrelation (predictability) in breeding habitat quality^71^. In these circumstances, social information may provide an efficient and easy way for individuals to assess and select where to settle^85^. However, drastic changes in adult survival could have decoupled habitat selection and quality (see above), so declining populations still maintaining relatively high densities of conspecifics may become ecological traps for local recruits, probably buffering them from steeper decline^86^, but reinforcing over time their deceptive appearance of high-quality sites. Inverse density-dependent dispersal also raises concerns about the population viability of sparse populations, which will lose locally-produced individuals via dispersal, increasing extinction probabilities^87^. Although all these results suggest that dispersal behaviours could depart from which would be ideal for population performance^88^, more information on the balance between immigration and emigration is needed^89,90^.

If emigration and immigration are governed by the same ‘avoid low density’ strategy, the speed with which the species can shift its range can be greatly reduced^91^. Our results indicate that dispersal from the natal site (emigration) is inversely related to population density. Recruitment patterns (immigration) also seem to be positively influenced by the presence and abundance of conspecifics, as the spatial distribution of territories explains both territory persistence and the establishment of new pairs^44,92^. Thus, Egyptian vultures colonize apparently appropriate empty areas very slowly in spite of being within the frequent range of dispersal distances, so recruits seem to be perceiving them as unsuitable (perceptual traps^93,94^). The fact that recently colonized areas are mostly situated in the proximity of healthy populations agrees with the distance-decaying dispersal distribution reported here, as well as with the higher tendency to emigrate from stable populations. It is also important to highlight that dispersal not only have effects on the spatial dynamics of (meta)populations, but the fitness payoffs of dispersal may be affected by such dynamical properties in an eco-evolutionary feedback loop^16,95,96^. As a consequence, dispersal may be selected against if moving becomes too costly due to habitat fragmentation or if occupied patches become too scarce^97–99^. Such a phenomenon could have occurred with the closely related bearded vulture in Spain and France, which was confined to the Pyrenees after intense human persecution and has not been able to leave this mountain range without human assistance despite significant population growth in recent decades^100^. In this way, if local extinctions continue to fragment Spanish and French populations of Egyptian vultures, evolutionary changes at the individual level could result in a profound discrepancy between dispersal behaviours and the levels of movement needed to maintain metapopulation processes^88^, thus exacerbating extinction probabilities.

### Perspectives

Although Euclidean distances are a fundamental characteristic of dispersal^13^, they only offer a basic description. Dispersal is a behavioural process that has a beginning (emigration from a site), an intermediate stage of movement (transience) and an end (immigration or settlement), and studying these three phases, their interdependence, and the different factors that come into play across them have been remarked as important to understand the complexity of dispersal dynamics^5,15,17^. The transience stage of Egyptian vultures lasts several years, during which birds may wander over long distances visiting several communal roosts as well as territories occupied by breeding pairs (Fig. S4^101^). Although the function of these extensive movements is unknown, it is likely that they were used at least in part to gather information on potential mates and settlement areas^71,102,103^, eventually determining natal dispersal distances. Interestingly, theory indicates a great propensity to invest in information acquisition to take informed dispersal decisions when the environment is predictable and prospecting costs are low^104^. However, Egyptian vultures seem to pay mortality costs just before settling as breeders^77^, suggesting that prospecting may not be so affordable. Although prospecting length may decrease with mortality risks, even in these circumstances some long-distance dispersal movements are expected to persist^105^. Indeed, dispersal patterns may reflect different dispersal strategies across stages and switches between movement modes during transience that are likely determined by the way particular phenotypes accumulate experience and respond to environmental conditions on the move^82,106,107^. All these aspects open exciting research avenues that will refine our understanding of dispersal in long-lived territorial birds.

## Methods

### Study system and data collection

We analysed natal dispersal distances of Egyptian vultures from six populations with contrasting densities and trends in Spain and France (Table 3), where the bulk of the European population is concentrated^108^. Egyptian vultures are medium-sized (~ 2 kg) obligate scavengers, which have declined sharply through its range^108^. They are territorial breeders that actively defend their breeding cliffs from conspecifics. However, during their long pre-adult stage, they lead a nomadic and social lifestyle. Pre-breeders typically roost communally near predictable food sources such as landfills, supplementary feeding stations, and large dumps of dead livestock, often performing large-scale movements between temporary roosting sites^109^. Median age of first breeding is at 7 years in peninsular Spain^33^, considerably later than in other raptors with similar size^75,110^. As typically occurs in species with a slow pace of life, Egyptian vultures have low fecundity (0–2 fledglings per breeding attempt). Mainland populations from Western Europe are typically long-distance migrants wintering in the sub-Saharan Sahel region^111^.

**Table 3:** Description of the six study populations of Spain and France.

Population	Period	Size	Density	Trend^b^	No. ring	No. rec	Age rec	Dispersal distance
Andalusia	2000–2015	25	10 (6–20)	− 4.8D	189	18	7.4 (5–11)	60.3 (0–546.9)
Bizkaia	2000–2015	20	67 (36–75)	0.3S	137	12	8.3 (5–13)	18.5 (8–551.9)
Ebro	1986–2015	60	49 (17–65)	− 3.1D	811	45	8.4 (3–24)	24.1 (0–272.4)
France SE	1997–2015	20	9 (2–11)	0.3S	263	35	6.6 (3–16)	78.5 (13.3–656.2)
France Pyrenees^a^	2008–2015	70	–	–	113	2	–	–
Segovia	2003–2015	30	31 (19–47)	− 2.1D	118	19	7.1 (5.10)	41.2 (5.5–200.8)

The study period, the size (approximate no. of breeding pairs), the density (median and range of the number of breeding pairs in a buffer of median natal dispersal distance around the natal nest), the annual percentage of growth rate (D and S denote declining and stable populations, respectively), the number of fledglings ringed during the study period (No. ring), the number of fledglings recruited as first-time breeders (No. rec.), age at recruitment (Age rec, mean and range, in years), and the dispersal distance (median and range, in kilometres) are shown. Note that only birds ringed until 2015 were considered, as our study finalized in 2018 and the youngest birds recruited when 3-year old.

^a^The few birds recruited as breeders were not sexed, so these birds were excluded from the analyses.

^b^The annual percentage of growth rate was calculated as $(({N}_{t}/{N}_{0} )^{1/(t-t0)}-1)\times 100$, where *N*_*0*_ and *N*_*t*_ are population sizes at times *t0* and *t*, respectively.

Between 1986 and 2015, we marked 1613 fledglings in six study areas (see Table 3 for details). Between 1986 and 2018, territories of each population were visited several times from March to September to check for the presence of birds, identify ringed individuals, and record breeding parameters. We also thoroughly inspected all cliffs with suitable nesting sites, so that virtually all pairs in each study area were located each year. Nests were accessed between June and August using conventional climbing techniques, and fledglings were marked with both metal and plastic rings with unique alpha-numeric codes or a combination of three coloured rings, making it possible for birds to be individually identified in the field with spotting scopes. Nestlings were measured, weighted, and most of them bled, but since data were collected as part of different projects, sampling protocols were not always consistent. This is particularly problematic for proxies of fledgling growth stage which were used to estimate body condition and hatching date. Thus, analyses were performed at two spatial scales, one at the regional level examining the effect of common predictors, and another at the local scale (in the Ebro valley population, from which we have the longest monitoring period and the largest marking effort), adding predictors for which sampling protocols were standardized (see “Predictor variables” below). Reencounter data used here came from our systematic population surveys, as well as from breeding birds observed opportunistically anywhere in Spain and France, mainly by forest rangers and naturalists.

The probability of detecting a marked bird that is alive, i.e. annual resighting probability, increased over the lifetime of Egyptian vultures in our study populations. It was very low during the first two years of life because birds often spend this period in Africa. It increased during the rest of the pre-breeding stage, but still with a high number of live birds not detected due to their nomadic lifestyle, and was typically very high (> 0.9) once birds recruited as breeders^46,48,77^.

### Predictor variables

At the regional scale, we used as environmental variables local population densities and trends. As an index for natal population density we used the total number of occupied territories within a buffer of 48,000 m of the nest of birth (median dispersal distance, see “Results”), calculated from the national census of Egyptian vultures carried out in 2008 for Spain^112^ and from unpublished information for France. We used these data for Spain because there were Egyptian vultures breeding outside our study areas, and therefore we did not have annual information on density within that buffer for all nests from which birds dispersed. When population density was calculated on the basis of the previous Spanish national census carried out in 2000^113^ our findings were qualitatively similar (“Results” not shown). For population trends, two categories were used according to observed population trajectories during the study period: declining and stable populations (see Table 3 for details). The individual predictors at this scale were sex, age at recruitment and brood size at fledgling. Birds from most populations were sexed by molecular techniques except for Bizkaia and France, whose birds were sexed by copulatory behaviour. At the Ebro valley local scale, natal population density was the unique environmental variable. In addition to the individual variables used at the whole scale, the following predictors were used: (1) birth date, as estimated from primary length at ringing^114^; (2) hierarchy rank within the brood (three categories: one fledgling and first or second fledgling from two-chick broods); (3) body condition at ringing time, as estimated from the residuals of an OLS regression of log-body mass on log-primary length (F_1,39_ = 14.77, P < 0.001, R^2^ = 0.27).

### Analytical and statistical procedures

We defined natal dispersal as the Euclidean distance between the birth site and the nest site where birds attempted to breed for the first time. We analysed log-transformed dispersal distances (logdist = log(dist + 1)) with linear models. Prior to fitting the models, we did some exploratory data analyses to visualize the distribution and structure of the data. In this step, we also explored if logarithmic or quadratic effects could potentially produce more robust inference, but linear terms always outperformed their non-linear counterparts. Two sets of candidate models were constructed, one for each spatial scale. Exploratory analyses and graphs suggested that some interactions between predictors could be operating in a sex-specific way at the Ebro valley local scale, so we additionally built linear models for each sex separately at this scale. For the analysis at the regional scale, population identity of origin was initially fitted as the random intercept in linear mixed models to control for multiple observations from the same study plot. However, population ID variance was estimated as zero in some models that included population density as a fixed predictor. Among-group variance was also estimated as zero for all random intercept models with Year identity as a random term, including the null model with no fixed effects. This indicated that all observed variance came from among-observation (residual) variance and virtually none from among-year variation. Thus, we proceeded with linear models without random effects and tested for spatial dependence in model residuals (see below). All combinations of main effects and two-way interactions were fitted, beginning with a global model and trying to reduce model complexity.

Before fitting the models, we centred the predictors by subtracting the mean, and assessed multicollinearity by calculating both overall and individual collinearity measures^115^. In all cases, values were sufficiently low (e.g. variance inflation factors VIF < 1.2) for collinearity to be of limited concern. We fitted candidate models using maximum likelihood (ML) estimation to allow model comparison. Model selection was based on Akaike’s Information Criterion corrected for sample size (AICc), and the relative fit of each model was ranked according to AICc differences (ΔAICc; Burnham and Anderson^116^). Following standard procedures, we calculated the Akaike weight for each candidate model (*w*_*i*_) as the relative strength of evidence, i.e., the probability of model *i* being the best-approximating model from the entire set of candidate models, and evidence ratios of the best models as the ratio of model weights. When there was no clear support for the highest-ranked model (less than 2 AICc points with respect to the second-ranked one), we used model averaging procedures to compute estimates of regression parameters. The relative importance of each predictor (RVI) was assessed by summing up the Akaike weights over the highest-ranked models including that predictor^117^. We used Moran’s I and semivariograms to evaluate the spatial pattern in the residuals from the top-ranked models^118^.

We used ArcGIS 10.5 (Environmental Systems Research Institute, Redlands CA) for spatial data management and calculations (dispersal distances, population density). All statistical analyses were performed in R 3.5.0.^119^. Multicollinearity was diagnosed with the package mctest^115^. For the most parsimonious models derived from AICc values, we inspected the scaled residuals using the package DHARMa^120^ and used standard methods for model fit diagnosis.

### Ethic statements

Bird-handling procedures and bird marking were authorized by Centre de Recherches sur la Biologie des Populations d'Oiseaux (CRBPO)—CNRS/MNHN (France), Gobierno de Aragón and Gobierno de Navarra (Ebro valley), Diputación Foral de Bizkaia (Bizcaia), Junta de Andalucía (Andalusia), and Gobierno de Castilla y León (Segovia), and following the protocols approved by the Ethic Committee of CSIC (CEBA-EBD-12-56).

## Supplementary Information

Supplementary Information
