# Supplementary material for: Phenotypic and environmental correlates of natal dispersal in a long-lived territorial vulture
Source: Sci Rep. 2021 Mar 8;11:5424. doi: 10.1038/s41598-021-84811-8 (PMC7970891; doi:10.1038/s41598-021-84811-8)

**PHENOTYPIC AND ENVIRONMENTAL CORRELATES NATAL DISPERSAL IN A LONG-LIVED TERRITORIAL VULTURE**

David Serrano^1^*, Ainara Cortés-Avizanda^1,2^, Iñigo Zuberogoitia^3^, Guillermo Blanco^4^, José Ramón Benítez^5^, Cecile Ponchon^6^, Juan Manuel Grande^7^, Olga Ceballos^8^, Jon Morant^9^, Eneko Arrondo^1, 10^, Jabi Zabala^11^, Eugenio Montelío^12^, Enrique Ávila^5^, José L. González del Barrio^4^, Bernardo Arroyo^13^, Óscar Frías^4^, Erick Kobierzycki^14^, Rafael Arenas^15^, José Luis Tella^1^ and José Antonio Donázar^1^

^1^Dept Conservation Biology, Estación Biológica de Doñana (EBD-CSIC), Avda. Americo Vespucio 26, E-41440 Sevilla, Spain. ^2^Animal Ecology and Demography Unit, IMEDEA (CSIC-UIB), C Miguel Marques 21, E-07190, Esporles, Mallorca, Spain. ^3^Estudios Medioambientales Icarus S.L. C/ San Vicente, 8. 6 ª Planta. Dpto 8. Edificio Albia I. E- 48001 Bilbao, Spain. ^4^Dept of Evolutionary Ecology, Museo Nacional de Ciencias Naturales (MNCN-CSIC), José Gutiérrez Abascal 2, E-28006 Madrid, Spain. ^5^Agencia Medioambiente & Agua, Linea Geodiversidad & Biodiversidad, E-41092 Seville, Spain. ^6^Conservatoire d’espaces naturels Provence-Alpes-Côte d’Azur, Maison de la Crau 2 place Léon Michaud,13310 Saint Martin de Crau, France. ^7^INCITAP-CONICET-UNLPam/FCEyN-UNLPam, Avda. Uruguay 151, Santa Rosa 6300, La Pampa, Argentina. ^8^UGARRA, Avda. Carlos III 1, 31002, Pamplona, Spain. ^9^Department of Ornithology, Aranzadi Sciences Society, Zorroagagaina 11, E-20014 Donostia-S. Sebastián, Spain. ^10^Dept of Applied Biology, Miguel Hernández University, Avda. de la Universidad, s/n, E-03202 Elche, Alicante, Spain. ^11^Sebero Otxoa 45 5 B. E-48480 Arrigorriaga, Biscay, Spain. ^12^Consultora CMC Sistemas de Mejora S.L. C/Vara de Rey, 48, Logroño, Spain. ^13^Caidos 4, E-19264 Alboreca, Guadalajara, Spain. ^14^19 rue de la poste, E-64800 Bruge , France. ^15^Delegación Territorial Córdoba CAGPDS, Tomás de Aquino, 7 planta, E-14071 Córdoba, Spain.

* e-mail: serrano@ebd.csic.es

**Table S1.** 95% set of linear models (minimum number of models whose cumulative Akaike weights sum to 0.95) for the effect of intrinsic and extrinsic variables on natal dispersal distances of Egyptian vultures across all study areas (region scale). Predictors: sx: sex; dn: density; tr: population trend; ag: age. “+” and “*” denote additive and multiplicative effects respectively. Null: intercept only model. Number of parameters (K), AICc, differences in AICc (ΔAICc), loglikelihood (logLik), Akaike weights (wi), cumulative weights (Cwi), and adjusted R-squared (R^2^) are shown. The null model (only intercept) is also shown.

| Model | K | AICc | ΔAICc | logLik | wi | Cwi | R^2^ |
| --- | --- | --- | --- | --- | --- | --- | --- |
| sx + dn + tr + sx*dn + dn*tr | 6 | 370.40 | 0.00 | -177.72 | 0.18 | 0.18 | 0.41 |
| sx + dn + tr + ag + sx*dn + dn*tr + sx*ag | 8 | 371.21 | 0.81 | -175.82 | 0.12 | 0.31 | 0.43 |
| sx + dn + tr + ag + sx*dn + dn*tr | 7 | 371.48 | 1.08 | -177.12 | 0.11 | 0.41 | 0.42 |
| sx + dn + tr + ag + sx*dn + dn*tr + dn*ag | 8 | 372.33 | 1.93 | -176.38 | 0.07 | 0.48 | 0.42 |
| sx + dn + tr + sx*dn + dn*tr + sx*tr | 7 | 372.43 | 2.03 | -177.59 | 0.07 | 0.55 | 0.41 |
| sx + dn + tr + ag + sx*dn + dn*tr + sx*tr + sx*ag | 9 | 373.10 | 2.70 | -175.58 | 0.05 | 0.60 | 0.43 |
| sx + dn + tr + ag + sx*dn + dn*tr + sx*ag + dn*ag | 9 | 373.11 | 2.71 | -175.58 | 0.05 | 0.65 | 0.43 |
| sx + dn + tr + ag + sx*dn + dn*tr + sx*tr | 8 | 373.56 | 3.16 | -176.99 | 0.04 | 0.68 | 0.42 |
| sx + dn + tr + ag + dn*tr + sx*ag | 7 | 373.64 | 3.23 | -178.19 | 0.04 | 0.72 | 0.40 |
| sx + dn + tr + dn*tr | 5 | 374.21 | 3.81 | -180.75 | 0.03 | 0.75 | 0.38 |
| sx + dn + tr + ag + sx*dn + dn*tr + sx*tr + dn*ag | 9 | 374.30 | 3.90 | -176.18 | 0.03 | 0.77 | 0.42 |
| sx + dn + tr + ag + dn*tr | 6 | 374.70 | 4.30 | -179.87 | 0.02 | 0.79 | 0.39 |
| sx + dn + tr + ag + sx*dn + dn*tr + sx*tr + sx*ag + dn*ag | 10 | 374.98 | 4.57 | -175.31 | 0.02 | 0.81 | 0.43 |
| sx + dn + tr + sx*dn | 5 | 375.07 | 4.67 | -181.18 | 0.02 | 0.83 | 0.37 |
| sx + dn + tr + ag + sx*dn + sx*ag | 7 | 375.19 | 4.78 | -178.97 | 0.02 | 0.85 | 0.40 |
| sx + dn + tr + ag + sx*dn + dn*ag | 7 | 375.85 | 5.45 | -179.30 | 0.01 | 0.86 | 0.39 |
| sx + dn + tr + ag + dn*tr + sx*ag + dn*ag | 8 | 375.86 | 5.45 | -178.14 | 0.01 | 0.87 | 0.41 |
| sx + dn + tr + ag + dn*tr + sx*tr + sx*ag | 8 | 375.91 | 5.51 | -178.17 | 0.01 | 0.88 | 0.41 |
| sx + dn + tr + ag + dn*tr + dn*ag | 7 | 376.11 | 5.71 | -179.43 | 0.01 | 0.89 | 0.39 |
| sx + dn + tr + ag + sx*dn | 6 | 376.12 | 5.71 | -180.58 | 0.01 | 0.90 | 0.38 |
| sx + dn + tr + dn*tr + sx*tr | 6 | 376.18 | 5.78 | -180.61 | 0.01 | 0.91 | 0.38 |
| sx + dn + tr + ag + sx*dn + sx*ag + dn*ag | 8 | 376.48 | 6.08 | -178.45 | 0.01 | 0.92 | 0.40 |
| sx + dn + tr + ag + dn*tr + sx*tr | 7 | 376.75 | 6.35 | -179.75 | 0.01 | 0.93 | 0.39 |
| sx + dn + tr + ag + sx*ag | 6 | 376.81 | 6.41 | -180.92 | 0.01 | 0.94 | 0.38 |
| sx + dn + tr + sx*dn + sx*tr | 6 | 377.27 | 6.87 | -181.15 | 0.01 | 0.94 | 0.37 |
| Null | 1 | 421.96 | 51.55 | -208.93 | 0 |  | 0 |

**Table S2.** Top-ranked linear models for the effect of intrinsic and extrinsic variables on natal dispersal distances of Egyptian vultures in the Ebro valley (local scale). Predictors: sx: sex; dn: density; ag: age; bs: brood size; hd: hatching date; sm: scaled mass. “+” and “*” denote additive and multiplicative effects respectively. Null: intercept only model. Number of parameters (K), AICc, differences in AICc (ΔAICc), loglikelihood (logLik), Akaike weights (wi), cumulative weights (Cwi), and adjusted R-squared (R^2^) are shown. The 40 highest-ranked models and the null model (only intercept) are shown.

| Model | K | AICc | ΔAICc | logLik | wi | Cwi | R^2^ |
| --- | --- | --- | --- | --- | --- | --- | --- |
| sx + dn + ag + sx*ag | 5 | 117.83 | 0.00 | -51.68 | 0.09 | 0.09 | 0.54 |
| sx + dn + ag | 4 | 118.32 | 0.49 | -53.30 | 0.07 | 0.16 | 0.50 |
| sx + dn | 3 | 118.91 | 1.08 | -54.90 | 0.05 | 0.21 | 0.45 |
| sx + dn + ag + bc | 5 | 119.56 | 1.73 | -52.54 | 0.04 | 0.25 | 0.52 |
| sx + dn + ag + bs + sx*ag | 6 | 119.61 | 1.78 | -51.11 | 0.04 | 0.28 | 0.55 |
| sx + dn + bc | 4 | 119.72 | 1.89 | -54.00 | 0.03 | 0.32 | 0.48 |
| sx + dn + ag + bc + sx*ag | 6 | 119.78 | 1.95 | -51.19 | 0.03 | 0.35 | 0.55 |
| sx + dn + ag + hd + sx*ag | 6 | 119.98 | 2.15 | -51.29 | 0.03 | 0.38 | 0.55 |
| sx + dn + ag + bs | 5 | 120.35 | 2.51 | -52.94 | 0.03 | 0.40 | 0.51 |
| sx + dn + ag + sx*dn + sx*ag | 6 | 120.40 | 2.56 | -51.50 | 0.02 | 0.43 | 0.54 |
| sx + dn + ag + hd | 5 | 120.59 | 2.76 | -53.06 | 0.02 | 0.45 | 0.50 |
| sx + dn + ag + sx*dn | 5 | 120.83 | 3.00 | -53.18 | 0.02 | 0.47 | 0.50 |
| sx + dn + hd | 4 | 121.11 | 3.28 | -54.70 | 0.02 | 0.49 | 0.46 |
| sx + dn + bs | 4 | 121.19 | 3.36 | -54.74 | 0.02 | 0.50 | 0.46 |
| sx + dn + sx*dn | 4 | 121.23 | 3.39 | -54.76 | 0.02 | 0.52 | 0.46 |
| sx + dn + bc + sx*dn | 5 | 121.52 | 3.68 | -53.52 | 0.01 | 0.53 | 0.49 |
| sx + dn + ag + bc + sx*dn | 6 | 121.65 | 3.82 | -52.13 | 0.01 | 0.55 | 0.53 |
| sx + dn + ag + hd + bs + sx*ag | 7 | 121.87 | 4.04 | -50.69 | 0.01 | 0.56 | 0.56 |
| sx + dn + ag + bc + sx*dn + sx*ag | 7 | 122.01 | 4.18 | -50.76 | 0.01 | 0.57 | 0.56 |
| sx + dn + ag + bs + bc | 6 | 122.16 | 4.32 | -52.38 | 0.01 | 0.58 | 0.52 |
| sx + dn + ag + bs + bc + sx*ag | 7 | 122.18 | 4.35 | -50.84 | 0.01 | 0.59 | 0.56 |
| sx + dn + ag + bc + dn*bc | 6 | 122.23 | 4.40 | -52.42 | 0.01 | 0.60 | 0.52 |
| sx + dn + ag + hd + bc | 6 | 122.24 | 4.41 | -52.42 | 0.01 | 0.61 | 0.52 |
| sx + dn + hd + bc | 5 | 122.31 | 4.47 | -53.92 | 0.01 | 0.62 | 0.48 |
| sx + dn + ag + hd + sx*dn + sx*ag | 7 | 122.34 | 4.50 | -50.92 | 0.01 | 0.63 | 0.56 |
| sx + dn + ag + bc + sx*bc | 6 | 122.37 | 4.54 | -52.49 | 0.01 | 0.64 | 0.52 |
| sx + dn + ag + hd + bc + sx*ag | 7 | 122.38 | 4.55 | -50.94 | 0.01 | 0.65 | 0.55 |
| sx + dn + bs + bc | 5 | 122.41 | 4.57 | -53.97 | 0.01 | 0.66 | 0.48 |
| sx + dn + ag + hd + sx*ag + sx*hd | 7 | 122.41 | 4.58 | -50.96 | 0.01 | 0.66 | 0.55 |
| sx + dn + ag + bs + sx*dn + sx*ag | 7 | 122.44 | 4.61 | -50.97 | 0.01 | 0.67 | 0.55 |
| sx + dn + bc + dn*bc | 5 | 122.45 | 4.61 | -53.99 | 0.01 | 0.68 | 0.48 |
| sx + dn + ag + bc + sx*ag + dn*bc | 7 | 122.46 | 4.63 | -50.98 | 0.01 | 0.69 | 0.55 |
| sx + dn + bc + sx*bc | 5 | 122.47 | 4.64 | -54.00 | 0.01 | 0.70 | 0.48 |
| sx + dn + ag + bs + sx*ag + dn*bs | 7 | 122.53 | 4.69 | -51.01 | 0.01 | 0.71 | 0.51 |
| sx + dn + ag + hd + sx*hd | 6 | 122.63 | 4.80 | -52.62 | 0.01 | 0.72 | 0.55 |
| sx + dn + ag + bs + sx*ag + sx*bs | 7 | 122.69 | 4.85 | -51.09 | 0.01 | 0.72 | 0.51 |
| sx + dn + ag + hd + bs | 6 | 122.76 | 4.93 | -52.68 | 0.01 | 0.73 | 0.55 |
| sx + dn + ag + bc + sx*ag + sx*bc | 7 | 122.80 | 4.97 | -51.15 | 0.01 | 0.74 | 0.51 |
| sx + dn + ag + hd + sx*dn | 6 | 123.02 | 5.19 | -52.82 | 0.01 | 0.75 | 0.51 |
| sx + dn + ag + bs + sx*dn | 6 | 123.08 | 5.25 | -52.84 | 0.01 | 0.75 | 0.51 |
| Null | 1 | 137.43 | 19.60 | -66.56 | 0 |  | 0 |

**Table S3.** 95% set of linear models for the effect of intrinsic and extrinsic variables on natal dispersal distances of male Egyptian vultures in the Ebro valley (local scale). Predictors: dn: density; ag: age; bs: brood size; hd: hatching date; bc: body condition. “+” and “*” denote additive and multiplicative effects respectively. Null: intercept only model. Number of parameters (K), AICc, differences in AICc (ΔAICc), loglikelihood (logLik), Akaike weights (wi), cumulative weights (Cwi), and adjusted R-squared (R^2^) are shown. The 40 highest-ranked models and the null model (only intercept) are shown.

| Model | K | AICc | ΔAICc | logLik | wi | Cwi | R^2^ |
| --- | --- | --- | --- | --- | --- | --- | --- |
| dn + ag | 3 | 72.43 | 0.00 | -31.10 | 0.21 | 0.21 | 0.45 |
| dn | 2 | 74.21 | 1.77 | -33.47 | 0.09 | 0.30 | 0.31 |
| dn + ag + bc + dn*bc | 5 | 74.25 | 1.82 | -28.50 | 0.09 | 0.38 | 0.57 |
| dn + ag + dn*ag | 4 | 74.34 | 1.91 | -30.41 | 0.08 | 0.47 | 0.48 |
| dn + ag + hd | 4 | 74.44 | 2.01 | -30.45 | 0.08 | 0.54 | 0.48 |
| dn + ag + bc | 4 | 75.01 | 2.58 | -30.74 | 0.06 | 0.60 | 0.47 |
| dn + bc | 3 | 75.43 | 3.00 | -32.60 | 0.05 | 0.65 | 0.36 |
| dn + bc + dn*bc | 4 | 75.97 | 3.54 | -31.22 | 0.04 | 0.69 | 0.44 |
| dn + ag + hd + dn*hd | 5 | 76.33 | 3.90 | -29.54 | 0.03 | 0.72 | 0.52 |
| dn + hd | 3 | 76.38 | 3.95 | -33.08 | 0.03 | 0.75 | 0.34 |
| dn + ag + hd + bc | 5 | 77.06 | 4.63 | -29.90 | 0.02 | 0.77 | 0.51 |
| dn + ag + hd + dn*ag | 5 | 77.25 | 4.82 | -30.00 | 0.02 | 0.79 | 0.50 |
| dn + hd + bc | 4 | 77.45 | 5.02 | -31.96 | 0.02 | 0.80 | 0.40 |
| dn + ag + bc + dn*ag | 5 | 77.49 | 5.05 | -30.12 | 0.02 | 0.82 | 0.50 |
| dn + ag + hd + bc + dn*bc | 6 | 77.87 | 5.44 | -28.20 | 0.01 | 0.83 | 0.58 |
| dn + ag + bc + dn*bc + ag*bc | 6 | 77.90 | 5.47 | -28.22 | 0.01 | 0.85 | 0.58 |
| dn + ag + bc + dn*ag + dn*bc | 6 | 78.08 | 5.65 | -28.31 | 0.01 | 0.86 | 0.57 |
| dn + ag + hd + ag*hd | 5 | 78.16 | 5.73 | -30.45 | 0.01 | 0.87 | 0.48 |
| dn + ag + bc + ag*bc | 5 | 78.64 | 6.21 | -30.70 | 0.01 | 0.88 | 0.47 |
| dn + ag + bs | 5 | 78.75 | 6.31 | -30.75 | 0.01 | 0.89 | 0.46 |
| ag | 2 | 79.04 | 6.61 | -35.89 | 0.01 | 0.90 | 0.14 |
| dn + hd + bc + dn*bc | 5 | 79.19 | 6.76 | -30.97 | 0.01 | 0.91 | 0.45 |
| dn + hd + dn*hd | 4 | 79.37 | 6.94 | -32.92 | 0.01 | 0.91 | 0.35 |
| Null | 1 | 79.73 | 7.30 | -37.57 | 0.01 | 0.92 | 0.00 |
| dn + ag + hd + dn*hd + ag*hd | 6 | 79.88 | 7.45 | -29.21 | 0.01 | 0.92 | 0.54 |
| dn + ag + hd + bc + dn*hd | 6 | 80.25 | 7.82 | -29.39 | 0.00 | 0.93 | 0.53 |
| dn + bs | 4 | 80.39 | 7.95 | -33.43 | 0.00 | 0.93 | 0.31 |
| dn + ag + hd + dn*ag + dn*hd | 6 | 80.52 | 8.09 | -29.53 | 0.00 | 0.93 | 0.52 |
| dn + ag + hd + bc + dn*ag | 6 | 80.58 | 8.15 | -29.56 | 0.00 | 0.94 | 0.52 |
| dn + ag + bs + dn*ag | 6 | 80.73 | 8.30 | -29.63 | 0.00 | 0.94 | 0.52 |
| dn + hd + bc + hd*bc | 5 | 80.77 | 8.34 | -31.76 | 0.00 | 0.94 | 0.41 |
| Dn + ag + hd + bc + ag*hd | 6 | 80.88 | 8.44 | -29.70 | 0.00 | 0.95 | 0.51 |

**Table S4.** 95% set of linear models for the effect of intrinsic and extrinsic variables on natal dispersal distances of female Egyptian vultures in the Ebro valley (local scale). Predictors: dn: density; ag: age; bs: brood size; hd: hatching date; bc: body condition. “+” and “*” denote additive and multiplicative effects respectively. Null: intercept only model. Number of parameters (K), AICc, differences in AICc (ΔAICc), loglikelihood (logLik), Akaike weights (wi), cumulative weights (Cwi), and adjusted R-squared (R^2^) are shown. The 40 highest-ranked models and the null model (only intercept) are shown.

| Model | K | AICc | ΔAICc | logLik | wi | Cwi | R^2^ |
| --- | --- | --- | --- | --- | --- | --- | --- |
| dn + hd + bc + dn*hd + hd*bc | 6 | 45.67 | 0.00 | -10.23 | 0.30 | 0.30 | 0.81 |
| dn + hd + dn*hd | 4 | 47.13 | 1.46 | -16.06 | 0.15 | 0.45 | 0.56 |
| dn + hd + bc + dn*hd | 5 | 47.97 | 2.31 | -14.17 | 0.10 | 0.55 | 0.66 |
| dn | 2 | 48.16 | 2.49 | -20.22 | 0.09 | 0.64 | 0.25 |
| ag + hd + bc + ag*hd + hd*bc | 6 | 49.89 | 4.22 | -12.34 | 0.04 | 0.67 | 0.74 |
| Null | 1 | 49.97 | 4.30 | -22.58 | 0.04 | 0.71 | 0.00 |
| dn + hd + bc + dn*hd + dn*bc | 6 | 50.08 | 4.41 | -12.44 | 0.03 | 0.74 | 0.74 |
| dn + bc | 3 | 50.16 | 4.49 | -19.54 | 0.03 | 0.77 | 0.31 |
| dn + ag | 3 | 50.60 | 4.93 | -19.76 | 0.03 | 0.80 | 0.29 |
| bc | 2 | 51.29 | 5.62 | -21.79 | 0.02 | 0.82 | 0.09 |
| dn + ag + hd + dn*hd | 5 | 51.31 | 5.64 | -15.84 | 0.02 | 0.84 | 0.57 |
| dn + hd | 3 | 51.50 | 5.84 | -20.21 | 0.02 | 0.85 | 0.25 |
| ag | 2 | 51.94 | 6.27 | -22.11 | 0.01 | 0.87 | 0.06 |
| dn + ag + hd + bc + dn*hd + hd*bc | 7 | 52.19 | 6.53 | -10.10 | 0.01 | 0.88 | 0.82 |
| dn + hd + bc + dn*hd + dn*bc + hd*bc | 7 | 52.30 | 6.63 | -10.15 | 0.01 | 0.89 | 0.82 |
| hd | 2 | 52.36 | 6.69 | -22.32 | 0.01 | 0.90 | 0.03 |
| dn + bc + dn*bc | 4 | 52.44 | 6.77 | -18.72 | 0.01 | 0.91 | 0.38 |
| ag + hd + ag*hd | 4 | 52.82 | 7.15 | -18.91 | 0.01 | 0.92 | 0.36 |
| hd + bc + hd*bc | 4 | 53.33 | 7.66 | -19.16 | 0.01 | 0.92 | 0.34 |
| dn + ag + hd + bc + dn*hd | 6 | 53.46 | 7.79 | -14.13 | 0.01 | 0.93 | 0.66 |
| dn + ag + bc + | 4 | 53.48 | 7.82 | -19.24 | 0.01 | 0.94 | 0.34 |
| dn + hd + bc | 4 | 54.03 | 8.37 | -19.52 | 0.00 | 0.94 | 0.31 |
| ag + bc | 3 | 54.06 | 8.39 | -21.49 | 0.00 | 0.95 | 0.12 |
| hd + bc | 3 | 54.29 | 8.62 | -21.61 | 0.00 | 0.95 | 0.11 |

**Table S5.** Moran’s I statistic used to evaluate spatial patterns in the residuals from the highest ranked models at the regional and local scale. We used a randomization test with 999 permutations to determine the probability of obtaining the observed value of *I.*

| Statistic | Observed rank | p |
| --- | --- | --- |
| *Regional scale* |  |  |
| -0.0070426 | 282 | 0.436 |
|  |  |  |
| *Local scale* |  |  |
| -0.062797 | 416 | 0.584 |

**Figure S1.** Model diagnostic plots for the best model obtained across all study areas. A: Histogram of log(dispersal distance +1); B: Residuals vs fitted values; C: QQ plot; D: scale-location; E: Cook’s distance plot; F: Residuals vs. Leverage plot. Note that the leverage plot indicated that there was a potential influence point (standardized residual <-3), but dropping it did not change the results qualitatively.


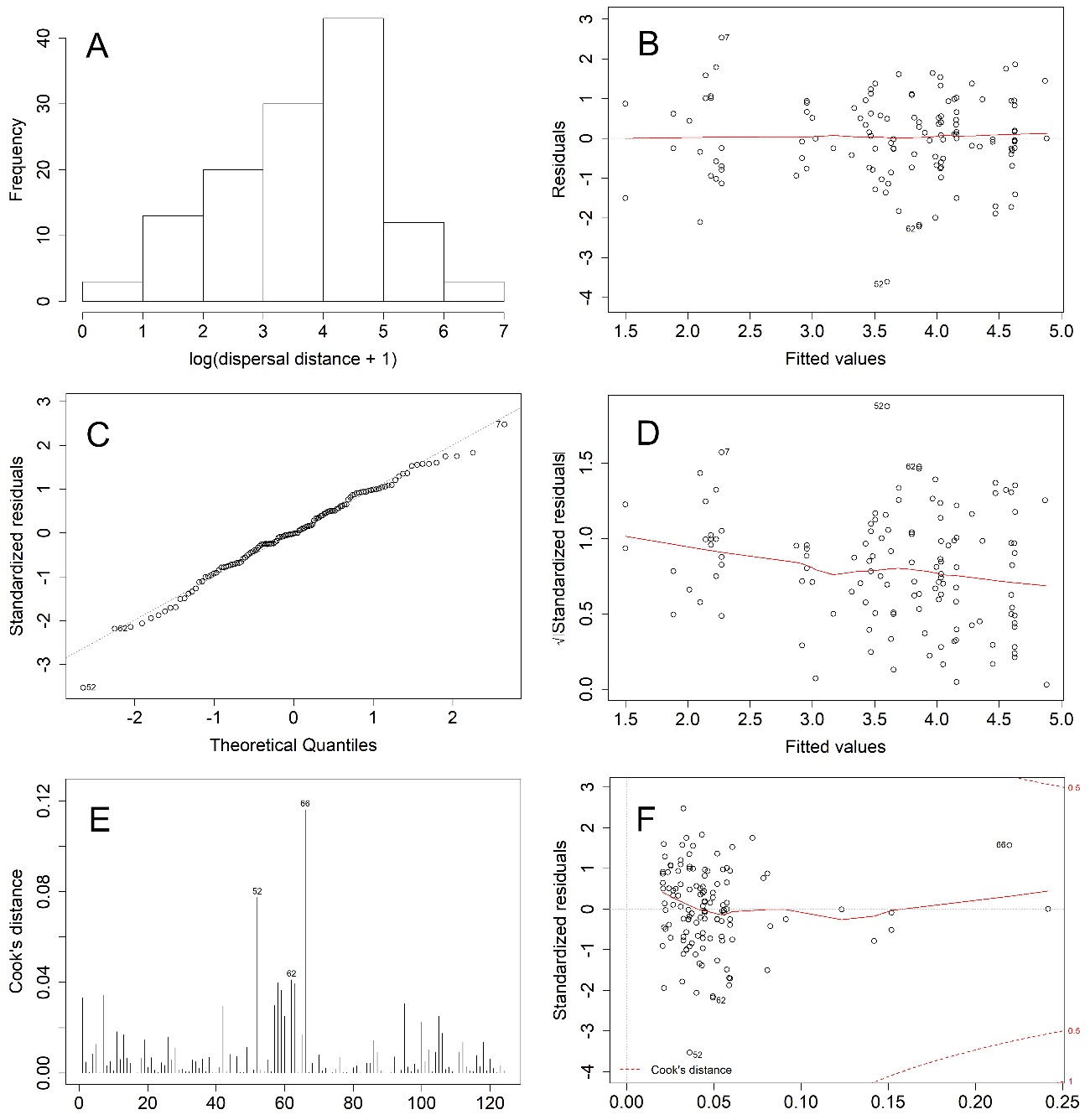


**Figure S2.** Model diagnostic plots for the best model obtained at the Ebro valley scale. A: Histogram of log(dispersal distance +1); B: Residuals vs fitted values; C: QQ plot; D: scale-location plot; E: Cook’s distance plot; F: Residuals vs. Leverage.


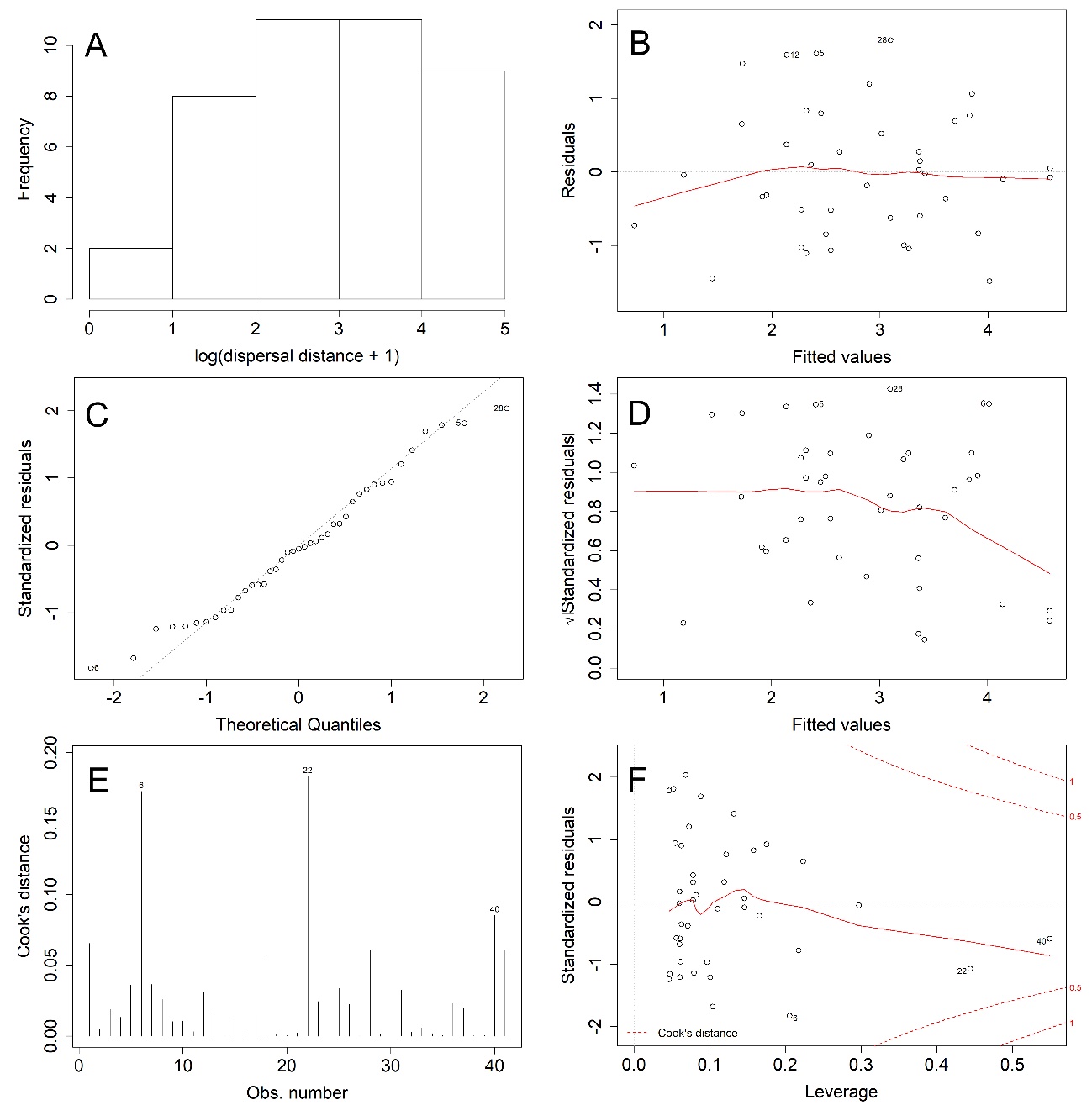


**Figure S3.** Semiovariograms showing the relationships between semivariance and distance between nests in which dispersing vultures were born at the regional (A) and local (B) scales.


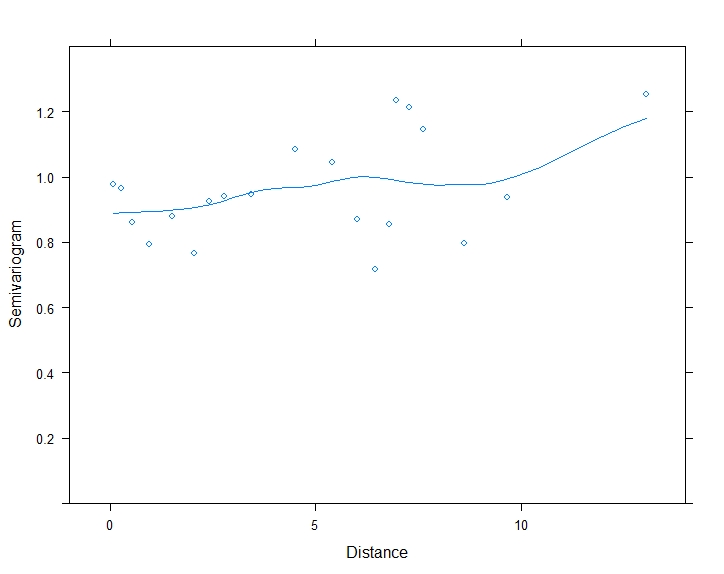


**A**


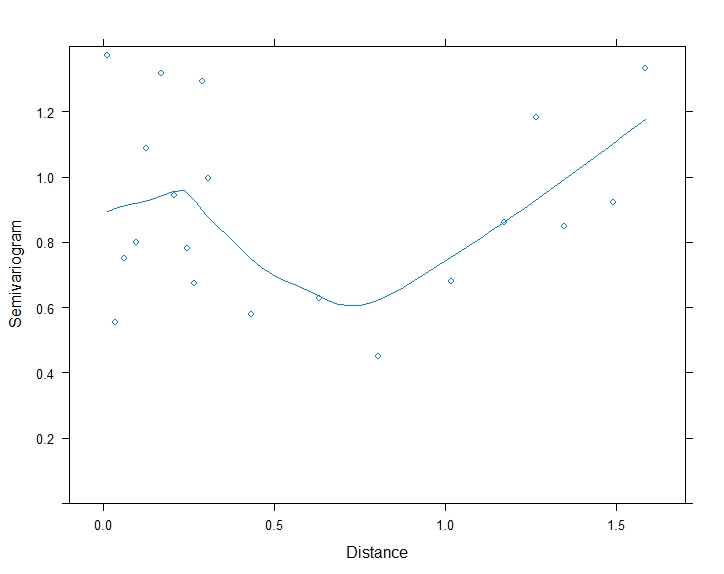


**B**

**Figure S4.** Movements during the pre-adult stage of 11 Egyptian vultures equipped with remote telemetry devices (PTTs with GPS) in Andalusia. Each colour represents a different individual. Movements during their stay in wintering quarters are not shown.


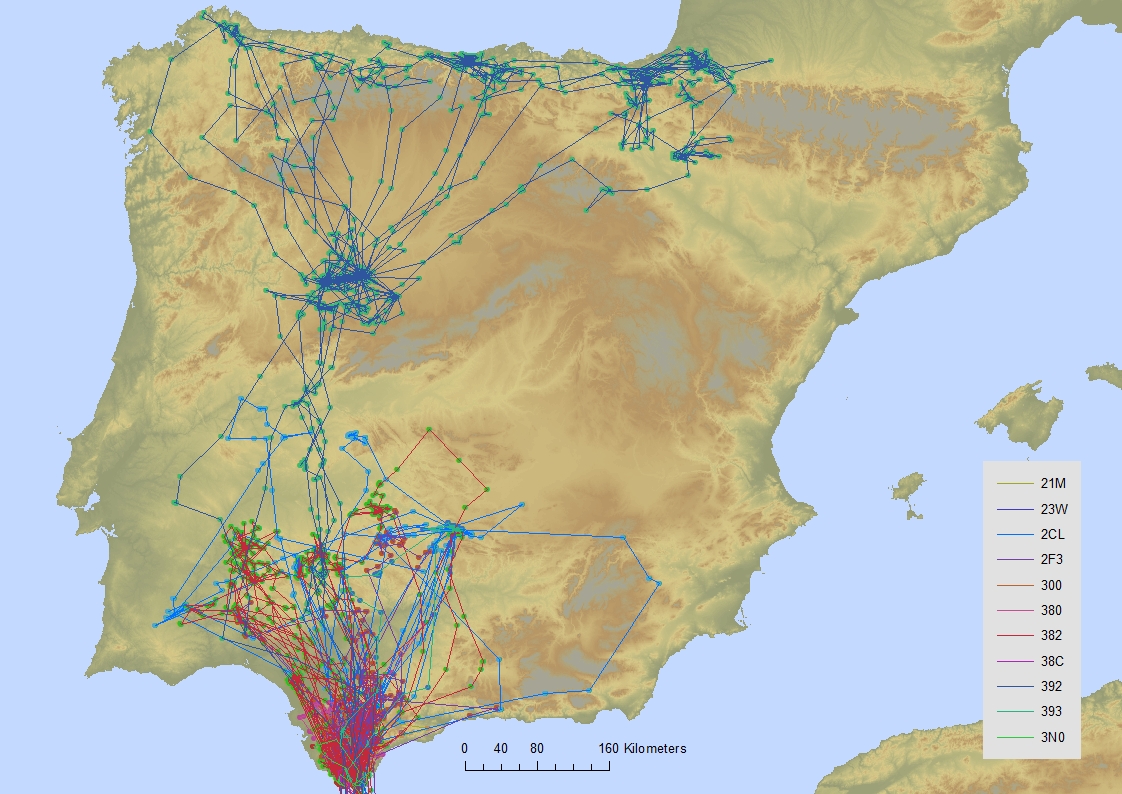

Supplement: Supplementary file 1 — Supplementary Information [file 41598_2021_84811_MOESM1_ESM.docx]
